# Concurrent Nephrotoxicity and Neurotoxicity Induced by Oral Valacyclovir in a Patient With Previously Normal Kidney Function

**DOI:** 10.7759/cureus.23693

**Published:** 2022-03-31

**Authors:** Ziad Abuhelwa, Azizullah Beran, Barat S Venkataramany, Bryan T Hinch, Ragheb Assaly

**Affiliations:** 1 Department of Medicine, University of Toledo, Toledo, USA

**Keywords:** hemodialysis, neurotoxicity, nephrotoxicity, acyclovir, valacyclovir

## Abstract

Drug-induced nephrotoxicity and neurotoxicity are commonly encountered problems in clinical practice. We describe a case of concurrent valacyclovir-induced nephrotoxicity and neurotoxicity in a 64-year-old man with no history of renal disease who developed acute renal injury and neurological symptoms after he received two weeks of the standard dose of oral valacyclovir for herpes zoster meningitis. His clinical condition improved significantly after the initiation of hemodialysis. Although nephrotoxicity due to intravenous infusion of valacyclovir and/or acyclovir is not uncommon, oral valacyclovir therapy is rarely associated with nephrotoxicity in patients with no history of renal insufficiency. Additionally, concurrent nephrotoxicity and neurotoxicity due to valacyclovir and/or acyclovir are rarely reported. Clinicians should be aware of these adverse events as immediate recognition and intervention are necessary to prevent morbidity.

## Introduction

Valacyclovir and/or acyclovir antiviral therapy has been the standard of care for the treatment of herpes viral infections. Oral valacyclovir is a prodrug that undergoes the first-pass intestinal and/or hepatic metabolism to produce active-moiety acyclovir and L-valine at a high bioavailability that is several times greater than that obtained from oral acyclovir [1]. Dosing adjustments proportionate to renal impairment may be required as patients with chronic renal disease are more susceptible to adverse effects [2]. Herein, we present a case of oral valacyclovir-induced nephrotoxicity and neurotoxicity in a man, with a previously normal kidney function, after receiving the standard dose of oral valacyclovir for the treatment of herpes zoster meningitis.

## Case presentation

A 64-year-old man presented to the emergency department due to progressively worsening generalized weakness and unsteady gait for five days. His family noted associated confusion and slurred speech. He reported no fever, chills, seizure, numbness, skin rash, or recent sick contact. Two weeks before his presentation, he was admitted for herpes zoster meningitis and was discharged on oral valacyclovir 1 g three times daily for 14 days. He had a past medical history of essential hypertension, paroxysmal atrial fibrillation, non-insulin-dependent type 2 diabetes mellitus, and hyperlipidemia. He denied tobacco, alcohol, or drug use. He was lethargic and oriented to person only on physical examination. He was afebrile and hemodynamically stable. He had positive flap asterixis but no facial drooping, tongue deviation, hemiparesis, or quadriparesis.

**Figure 1:**
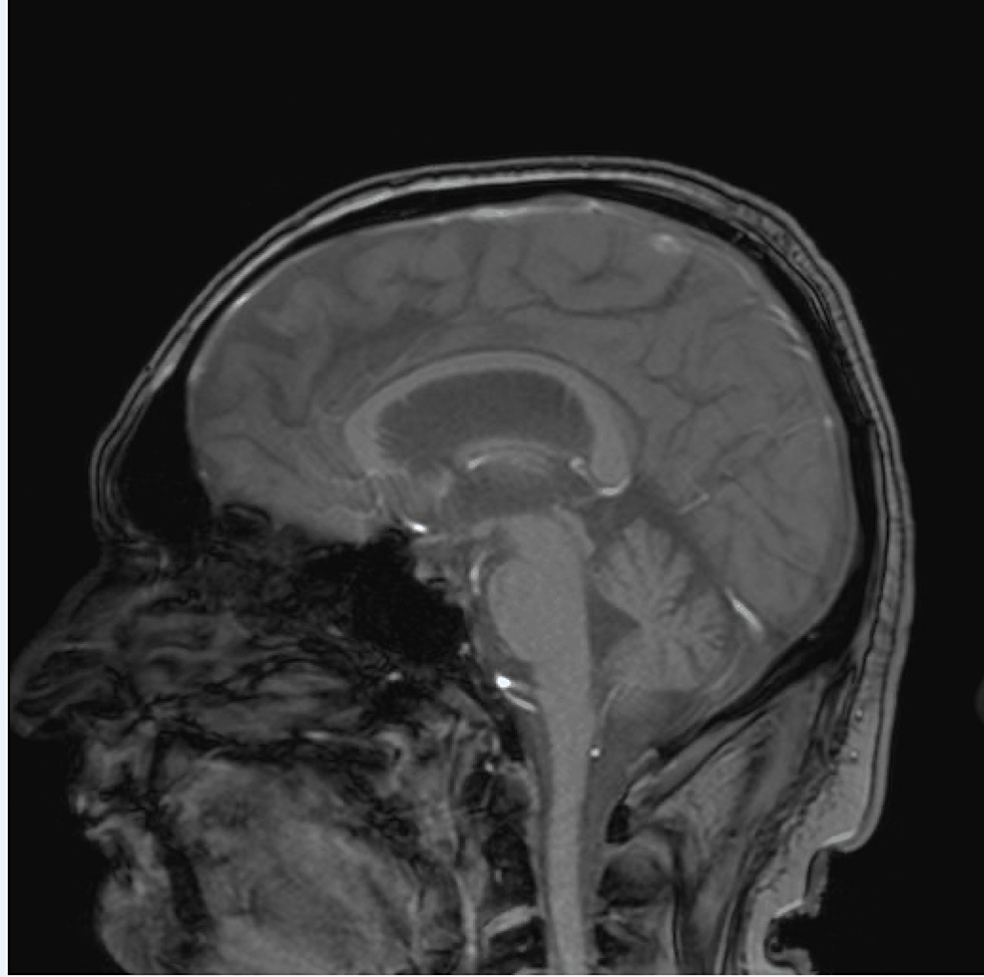
Brain MRI Sagittal view showing the cerebrum and cerebellum. Mild global parenchymal volume loss is noted.

Laboratory investigations on initial presentation are summarized in Table 1. A complete blood count showed mildly elevated white blood cells, but otherwise unremarkable. Serum chemistry showed hyponatremia, upper normal potassium level, mildly elevated phosphorus, elevated blood urea nitrogen of 67 mg/dL, and creatinine of 5.68 mg/dL. Further testing including acute hepatitis panel and human immunodeficiency virus was nonreactive. Serum C3 and C4 complements were within normal limits. Additionally, serum antineutrophil cytoplasmic antibodies and antiglomerular basement membrane antibodies were negative. Microscopic urine analysis showed six red blood cells with no casts, and the urine eosinophil test was negative. Renal ultrasound of both kidneys revealed preservation of the size with normal renal cortex echogenicity. Brain magnetic resonance imaging showed unremarkable findings (Figure 1). Routine electroencephalogram showed generalized slowing with no identified epileptiform discharges. Plasma acyclovir level was elevated at 6.5 µg/mL (normal therapeutic range: 0.40-2.0).

**Table 1:** Results of laboratory investigations on initial presentation c-ANCA: cytoplasmic antineutrophil cytoplasmic antibodies, p-ANCA: perinuclear antineutrophil cytoplasmic antibodies, anti-GMB: antiglomerular membrane antibodies, anti-HAV: antihepatitis A antibodies, anti-HBc: antihepatitis B antibody, HBs Ag: hepatitis B surface antigen, anti-HCV; antihepatitis C antibodies, HIV-1&2 Ag/Ab: human immunodeficiency virus 1 and 2 antigen and antibody.

Test	Test Result	Reference Range
White blood cells (×10E9/L)	12.3	4.0-11.0
Hemoglobin (mg/dL)	16.1	13.0-17.0
Platelets (×10E9/L)	306	150-450
Sodium (mmol/L)	122	134-146
Potassium (mmol/L)	5	3.5-5.0
Creatinine (mg/dL)	5.68	0.60-1.30
Blood urea nitrogen (mg/dL)	67	5.0-27.0
Phosphorus (mg/dL)	5.1	2.4-4.9
Calcium (mg/dL)	9.2	8.5-10.5
Albumin (g/dL)	3.6	3.2-5.3
Total protein (g/dL)	6.8	6.0-8.0
Uric acid (mg/dL)	5.9	2.6-7.2
C3 complement	121	86-184
C4 complement (mg/dL)	39	6.0-47.0
c-ANCA	Negative	Negative
p-ANCA	Negative	Negative
Anti-GBM	Negative	Negative
IgM anti-HAV	Negative	Negative
IgM anti-HBc	Negative	Negative
HBs Ag	Negative	Negative
Anti-HCV	Negative	Negative
HIV-1&2 Ag/Ab	Negative	Negative

**Figure 2:**
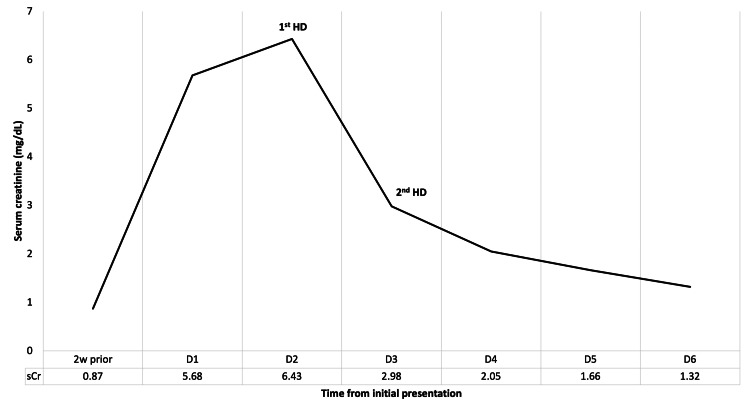
Serial changes of serum creatinine over time starting from two weeks prior to initial presentation until discharge sCr: serum creatinine, 2w prior: two weeks prior, D1-D6: day one to day six, HD: hemodialysis.

He was initially started on intravenous isotonic fluids with no clinical or laboratory improvement. On the following day, his creatine increased to 6.43 mg/dL and blood urea nitrogen to 77 mg/dL (Figure 2). An urgent temporary dialysis catheter was placed, and he received two sessions of hemodialysis over two consecutive days. After the second session, significant symptomatic improvement was noted. On day six of presentation, he was fully back to his baseline mental status with near normalization of kidney function as serum creatinine was 1.32 mg/dL and blood urea nitrogen was 20 mg/dL. The patient was discharged home with a close follow-up with his primary care physician.

## Discussion

Valacyclovir is a prodrug that inhibits viral deoxyribonucleic acid (DNA) polymerase and produces the active metabolites acyclovir and L-valine [1]. Valacyclovir- and/or acyclovir-induced nephrotoxicity has been described in the literature. Roberts et al. described a case of acute kidney injury after acute valacyclovir overdose in a relatively young man [3]. Sugimoto et al. reported a case of acute renal failure in an elderly man with known chronic kidney disease after five days of oral valacyclovir (1 g three times daily) administration [4]. Neurotoxicity has been described to a lower extent compared to nephrotoxicity. Chowdhury et al. reported a case of neurotoxicity in an end-stage renal disease patient after three days of intravenous acyclovir (750 mg twice daily) administration [5]. Combined valacyclovir- and/or acyclovir-induced nephrotoxicity and neurotoxicity at the same time have been rarely described. Krieble et al. described a case of combined acyclovir-induced nephrotoxicity and neurotoxicity in a newly diagnosed acquired immunodeficiency syndrome patient while receiving a high dose of intravenous acyclovir for disseminated herpes zoster infection [6]. Murakami et al. described another case of combined valacyclovir-induced nephrotoxicity and neurotoxicity in an elderly woman [7]. In our patient, even though he had normal kidney function and received the appropriate dosage of oral valacyclovir for herpes zoster meningitis, he developed both nephrotoxicity and neuropsychiatric adverse events at the same time.

The ability to cause crystal-induced nephropathy is well characterized in the literature as a mechanism for valacyclovir- and/or acyclovir-induced nephrotoxicity. Acyclovir is insoluble in the urine, which can lead to crystal nephropathy. However, this is more common with intravenous administration due to the poor bioavailability of oral acyclovir [8]. In rare situations, interstitial nephritis and direct tubular necrosis may result in renal impairment [7]. On the other hand, the underlying pathophysiology for valacyclovir- and/or acyclovir-induced neurotoxicity is not well elucidated. A study proposed that large doses of antiviral drugs can inhibit mitochondrial DNA polymerase, and the resultant cellular dysfunction triggers neurotoxicity [9].

The main treatment for valacyclovir- and acyclovir-induced nephrotoxicity and neurotoxicity is to stop the offending medications. Hemodialysis, hemofiltration, and hemodiafiltration are all extracorporeal procedures that are effective at removing acyclovir because of its small molecular weight (225 Da), limited protein binding rate, low steady-state volume of distribution, and high water solubility [10]. After two hemodialysis sessions, our patient's mental status and kidney function improved significantly.

## Conclusions

In conclusion, even in patients with normal kidney function, regular doses of oral valacyclovir can cause nephrotoxicity and neurotoxicity, as described in our case. Our case aims to bring awareness to this rare adverse event of valacyclovir. Clinicians should consider valacyclovir as a possible culprit of altered mental status and acute kidney injury even in the absence of chronic kidney disease. Monitoring acyclovir plasma levels may play a role in the diagnosis of neurotoxicity. These adverse events can respond well to hemodialysis.

## References

[1] Ormrod D, Goa K (2000). Valaciclovir: a review of its use in the management of herpes zoster. Drugs.

[2] Perazella MA (1999). Crystal-induced acute renal failure. Am J Med.

[3] Roberts DM, Smith MW, McMullan BJ, Sevastos J, Day RO (2011). Acute kidney injury due to crystalluria following acute valacyclovir overdose. Kidney Int.

[4] Sugimoto T, Yasuda M, Sakaguchi M (2008). Oliguric acute renal failure following oral valacyclovir therapy. QJM.

[5] Chowdhury MA, Derar N, Hasan S, Hinch B, Ratnam S, Assaly R (2016). Acyclovir-induced neurotoxicity: a case report and review of literature. Am J Ther.

[6] Krieble BF, Rudy DW, Glick MR, Clayman MD (1993). Acyclovir neurotoxicity and nephrotoxicity: the role for hemodialysis. Am J Med Sci.

[7] Murakami T, Akimoto T, Okada M (2018). Valacyclovir neurotoxicity and nephrotoxicity in an elderly patient complicated by hyponatremia. Drug Target Insights.

[8] Kitano A, Motohashi H, Takayama A, Inui KI, Yano Y (2015). Valacyclovir-induced acute kidney injury in Japanese patients based on the PMDA adverse drug reactions reporting database. Ther Innov Regul Sci.

[9] Lewis W, Dalakas MC (1995). Mitochondrial toxicity of antiviral drugs. Nat Med.

[10] Yang HH, Hsiao YP, Shih HC, Yang JH (2007). Acyclovir-induced neuropsychosis successfully recovered after immediate hemodialysis in an end-stage renal disease patient. Int J Dermatol.

